# P-109. A Phase I First-in-Human Study to Evaluate the Safety and Pharmacokinetic (PK) Properties of the Intravenously (IV) Administered OMN6, A Novel Antimicrobial Peptide Targeting *Acinetobacter baumannii* (AB), in Adults including Older Healthy Volunteers (HVs) Subjects

**DOI:** 10.1093/ofid/ofae631.316

**Published:** 2025-01-29

**Authors:** Bella Shusterman Muratov, Shelly Maximov, Yafit Stark, Jonathan Zazoun, Janna Michaeli, Peter Dogterom, Khalid Abd-Elaziz, Andrew Shorr, Moshik Cohen Kutner, Niv Bachnoff

**Affiliations:** Omnix Medical Ltd., Jerusalem, Yerushalayim, Israel; Omnix Medical Ltd., Jerusalem, Yerushalayim, Israel; Omnix Medical Ltd., Jerusalem, Yerushalayim, Israel; Omnix Medical Ltd., Jerusalem, Yerushalayim, Israel; Omnix Medical Ltd., Jerusalem, Yerushalayim, Israel; QPS Netherlands, Groningen, Groningen, Netherlands; QPS Netherlands, Groningen, Groningen, Netherlands; Medstar Washington Hospital Center, Not Applicable; Omnix Medical Ltd., Jerusalem, Yerushalayim, Israel; Omnix Medical Ltd., Jerusalem, Yerushalayim, Israel

## Abstract

**Background:**

*Acinetobacter baumannii* (AB) is an opportunistic Gram-negative pathogen ranked 1^st^ on the World Health Organization (WHO) Priority Pathogen list, that causes severe infections among elderly patients in intensive care units. Given the accelerating pace of global population aging, the burden of diseases including multi-drug resistant AB (MDRAB) infections is increasingly falling on elderly adults. Due to both elevated mortality rates associated with AB and the lack of effective treatments in older adults, there is a critical unmet need to develop new effective and safe anti-infectives. OMN6 is a novel, biochemically-engineered antimicrobial peptide, with a unique mechanism of action, which is being developed for the treatment of severe AB infections including carbapenem-resistant AB and MDRAB.

**Methods:**

The First in Human Phase 1 OMN6 clinical trial was a single center, double-blind, placebo-controlled, randomized, single ascending total daily dose study, conducted in healthy volunteers. 5 single ascending total daily doses were tested; 5 cohorts in adults aged 18-59 years, and 1 cohort of older adults aged ≥ 60 years. Adults aged 18-59 years received doses ranging from 7.5 to 100 mg OMN6 as a 3-hour single IV infusion, while 50 mg was administered to both age groups (N=8/cohort). Safety and tolerability assessments, and pharmacokinetic (PK) blood sampling occurred at pre-defined timepoints. All blood samples for PK evaluation were analyzed with a validated LC/MS/MS assay. PK parameters of 50 mg OMN6 in both age groups were compared.

**Results:**

No serious adverse events (SAEs) were reported in either adults or older adults and all dose levels were considered safe and well tolerated.

Upon a single infusion administration, mean PK results demonstrated dose-proportionality for C_max_ and near dose-proportionality for AUC_inf_. The OMN6 PK profiles in adults and older adults at the 50 mg dose indicated a similar pattern. The mean AUC_inf_ and C_max_ for adults and older adults, were 1289 and 1211 h*ng/mL; and 502 and 470 ng/mL, respectively.

**Conclusion:**

OMN6 had a favorable safety, tolerability, and PK profile that was similar in adults across all age groups. These positive results support advancing OMN6 to Phase 2 clinical trials, including subjects aged ≥ 60 years.

**Disclosures:**

**Andrew Shorr, MD, MPH, MBA**, Basilea: Advisor/Consultant|Basilea: Grant/Research Support

